# Genome-wide characterization of non-reference transposons in crops suggests non-random insertion

**DOI:** 10.1186/s12864-016-2847-3

**Published:** 2016-08-02

**Authors:** Bin Wei, Hanmei Liu, Xin Liu, Qianlin Xiao, Yongbin Wang, Junjie Zhang, Yufeng Hu, Yinghong Liu, Guowu Yu, Yubi Huang

**Affiliations:** 1 Maize Research Institute, Sichuan Agricultural University, Chengdu, 611130 China; 2 College of Life Science, Sichuan Agricultural University, Ya’an, 625014 China; 3 Beijing Genome Institute and the Key Laboratory of Genomics of the Minister of Agriculture, Shenzhen, 518083 China; 4 College of Agronomy, Sichuan Agricultural University, Chengdu, 611130 China

**Keywords:** Non-reference transposons, Genome-wide analysis, Crop, Non-random insertion, Stress response

## Abstract

**Background:**

Transposons (transposable elements or TEs) are DNA sequences that can change their position within the genome. A large number of TEs have been identified in reference genome of each crop(named accumulated TEs), which are the important part of genome. However, whether there existed TEs with different insert positions in resequenced crop accession genomes from those of reference genome (named non-reference transposable elements, non-ref TEs), and what the characteristics (such as the number, type and distribution) are. To identify and characterize crop non-ref TEs, we analyzed non-ref TEs in more than 125 accessions from rice (*Oryza sativa*), maize (*Zea mays*) and sorghum (*Sorghum bicolor*) using resequenced data with paired-end mapping methods.

**Results:**

We identified 13,066, 23,866 and 35,679 non-ref TEs in rice, maize and sorghum, respectively. Genome-wide characterization analysis shows that most of non-ref TEs were unique and non-ref TE classes shows different among rice, maize and sorghum. We found that non-ref TEs have a strong positive correlation with gene number and have a bias toward insertion near genes, but with a preference for avoiding coding regions in maize and sorghum. The genes affected by non-ref TE insertion were functionally enriched for stress response mechanisms in all three crops.

**Conclusions:**

These observations suggest that transposon insertion is not a random event and it makes genomic diversity, which may affect the intraspecific adaption and evolution of crops.

**Electronic supplementary material:**

The online version of this article (doi:10.1186/s12864-016-2847-3) contains supplementary material, which is available to authorized users.

## Background

Transposons are DNA sequences that can change their positions within the genome. Transposons were first discovered in maize by McClintock in the 1940s [1], and over the next several decades, transposons have been found in almost every plant and animal genome. Moreover, transposons are important components of crop genomes. For example, at least 35 % of the rice genome [2], 62 % of the sorghum genome [3], and nearly 85 % of the maize genome [4] is made up of transposable elements (TEs).

A scheme for the classification of transposons is based on transposition mechanisms, sequence similarities and structural relationships [5]. Transposons are divided into two classes: DNA transposons and RNA transposons (retrotransposons) [6]. Retrotransposons include the following three groups: Long terminal repeats (LTRs), which are flanked by long terminal repeats and encode reverse transcriptase; long interspersed elements (LINEs), which lack LTRs and are transcribed by RNA polymerase II; and short interspersed elements (SINEs), which also lack LTRs and are transcribed by RNA polymerase III. In addition, there are the helitrons, which are replicated by the ‘rolling-circle’ mechanism, and are therefore also called rolling-circle (RC) transposons. Transposons of theses classes are widely distributed and constitute major components of plant genomes. Additionally, TE superfamilies may be subdivided depending on their replication strategies in crops, such as LTR/*Copia*, LTR/*Gypsy*, DNA/*CMC-EnSpm*, DNA/*MULE-MuDR*, LINE/*L1* and RC/*Helitron*.

In recent years, we have gradually realized the importance of transposons in genome structure, function and evolution. As a fundamental function elements constituting the genomes, transposons are playing important roles in the formation and evolution of the DNA “jigsaw puzzle” structure. They are distributed nonrandomly in large genome and have a correlative relation between other function elements [7, 8]. Transposons not only affect plant genome structure but also play important roles in gene expression regulation [9]. Their activity can inactivate genes. Some transposons prefer insertion into genes or near gene flanking regions, leading to a mutation that affects gene function. This transposon activity can be engineered using appropriate vectors to produce artificial mutations in genes. For example, wrinkled peas result from a 0.8-KB transposon insertion in the SBE1 gene, the mechanism of which is similar to the mechanism for the corn *Ac/Ds* transposon family [10]. Transposon insertion can also positively or negatively alter gene expression levels. A classic example is the transposon insertion into intron 1 of the maize knotted1 gene, causing the expression in the leaves [11]. In additional, transposon insertions can also cause gene rearrangement and epigenetic silencing.

With the advance of high-throughput sequencing and data analysis technologies, researchers have been able to identify new transposon insertions in various species. A comparative genome analysis showed that 14 % of the genomic differences between Nipponbare and 9311 are the result of transposon insertion [12]. Naito et al. detected 1664 mPing transposon insertions by analyzing the genome resequencing data of 24 rice accessions [13]. Ewing and Kazazian analyzed data from the 1000 Genomes Project and presented their analysis of LINE-1 insertions in genomes that are not represented in the reference genome assembly [14]. Tian et al. analyzed sequencing data of 31 wild and cultivated soybeans and detected 34,154 new transposon insertions, which revealed the evolutionary trends of transposons in soybean [15]. The above studies demonstrate that transposons between accessions of the same species are markedly different, and these differences may play important roles in the evolution of species.

Rice, maize and sorghum are important cereal crops; all of their reference genomes are available. Many landrace accessions of these crops and improved and wild varieties have been resequenced using second-generation sequencing technology. Lai et al. resequenced six maize inbred lines, and 103 maize lines of teosintes, landraces and improved varieties were resequenced in the maize hapmap2 project (HapMapV2) [16]. Genome resequencing of 40 cultivated lines and ten wild lines of rice were completed, with an average depth of >15X [17]. Mace et al. resequenced 45 sorghum varieties with an average sequencing depth of 16–45X [18]. At the same time, many methods and tools have been used to identify new transposon insertions in resequenced accessions, which are inserted in different genomic locations from those of reference genome, and termed non-reference transposable elements (non-ref TEs). That is, non-ref TEs are not in the reference genome but in other resequenced accession genomes. RetroSeq introduced a method using pair-end reads mapping to reference genome and accumulated transposon database to do this. First, one end of the pair-end short reads are mapped to the reference genome, while the other paired reads are mapped to the transposons library; paired short reads will therefore overlap with potential transposon insertion sites. Second, transposons that pass aggregation analyses of all possible positions and filtering for depth coverage are designated as non-reference transposons [19]. Although transposons are major components of the genome, their exact functions and relevance in plant genomes have not been revealed. Genome resequencing of crop accessions can be used efficiently to identify and characterize Non-ref TEs. Comparing to the reference genome, Non-ref TEs have different insert positions in accessions.

In this study, resequencing data of 125 accessions for rice, maize and sorghum were collected, including wild, landrace and improved groups. Non-ref TEs were identified using pair-end read alignment to the reference genome and transposon databases separately. To characterize genome-wide non-ref TEs, we compared classes of non-ref TEs between both species and groups and analyzed the insertion location and affected genes. We found that the number, classification and distribution of non-ref TEs were different for each crop group and each accessions of the same species. In addition, non-ref TEs had an insertion preference for intergenic regions, avoiding coding regions. These observations suggest that transposon insertion is not a random event. Furthermore, the functional analysis of affected genes suggested that transposon insertion plays an important role in the adaptive evolution of crops.

## Results

### Identification of non-ref TE insertions

We used the RepeatMasker (Version: 3.3.0) [20] with the TE database library exacted from RepbaseUpdate to predict the accumulated TEs. The results of this analysis identified total lengths of 142,446,614 bp for TEs in rice, 1,585,325,106 bp for TEs in maize, and 434,877,678 bp for TEs in sorghum, comprising 37.22, 76.72 and 58.88 % of the three reference genomes, respectively.

To identify non-ref TEs in the next-generation sequencing data, we optimized a previously released pipeline [19] (see Methods). In 50 rice accessions, we identified 13,066 non-ref TEs, with an average of 261 non-ref TEs for each accession. A total of 23,866 non-ref TEs were identified in 30 maize accessions, with an average of 796 non-ref TEs. For 45 sorghum accessions, 35,679 non-ref TEs were discovered, with an average of 793 (Table 1 and Additional file 1: Table S4). According to their different evolutionary and domestication history, we divided them into three groups of improved, landrace and wild. The NPSPD (Average number of non-ref TEs per sample per depth) in the wild group was highest, followed by landraces. The NPSPD of the improved group was lowest in rice and sorghum (because the wild group of maize had only one accession, no comparison could be made). The results were consistent with the genetic differences between groups, which suggest the reliability of our approach for identifying non-ref TEs.

**Table 1:** Summary of the non-ref TEs in rice, maize, sorghum

Species	Groups	Sample size	Total reads	Raw data depth	Average depth	Non-ref TEs	Average non-ref TEs	NPSPD^a^	Average length^b^	% in genome^c^
*O. stativa*		50	3901075202	934.61	18.69	13066	261	0.28	24312832	0.064
	Improved	11	929384266	213.98	19.45	2744	249	1.17	5238995	0.014
	Landrace	29	2173501326	533.99	18.41	8914	307	0.58	16703160	0.044
	Wild	10	798189610	186.64	18.66	5175	518	2.77	9477388	0.025
*Z. mays*		30	3717985422	182.96	6.10	23866	796	4.35	41408468	0.020
	Improved	6	1079477218	52.24	8.71	9846	1641	31.41	17762074	0.009
	Landrace	23	2530119502	124.94	5.43	15628	679	5.44	26251683	0.013
	Wild	1	108388702	5.78	5.78	1798	1798	311.07	3150689	0.002
*S. bicolor*		45	7633325734	891.37	19.81	35679	793	0.89	53089501	0.072
	Improved	20	3574432140	397.59	19.88	19980	999	2.51	29721648	0.040
	Landrace	18	2678808170	325.89	18.10	15437	858	2.63	22776676	0.031
	Wild	7	1380085424	167.89	23.98	17941	2563	15.27	25363817	0.034

^a^NPSPD, Average number of Non-ref TEs per sample per depth

^b^Average length of non-ref TEs Length (bp)

^c^Average length of non-ref TEs (bp)/reference genome size (bp)

**Fig. 1:**
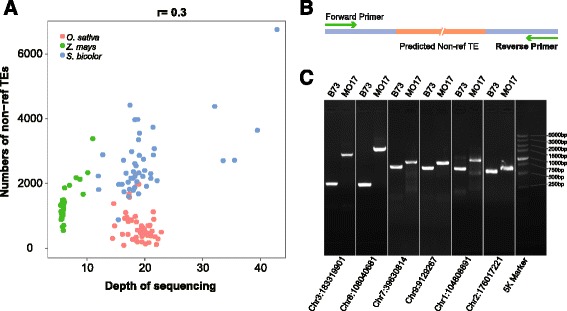
Identification of non-ref TEs in rice, maize and sorghum. **a** Correlation between sequence depth and numbers of non-ref TEs. **b** Diagram of primer design to validate target TE insertion events. **c** PCR-based validation of non-ref TEs insertion in maize

The sequencing depth of the accessions we studied ranged from 6X to 45X, of which the average depths were 18X, 6X and 20X for rice, maize and sorghum, respectively. For our method of mapping reads to identify non-ref TE positions, when the non-ref TEs were complete identified, high sequence depth should not increase the number of non-ref TEs. To determine whether the number of identified non-ref TEs was associated with accession sequencing depth, we calculated the Pearson correlation coefficient between sequencing depth and the number of non-ref TEs for all accessions. The results showed a Pearson correlation coefficient of 0.3, suggesting no obvious correlation between the two indices (Fig. 1a) and making our method reasonable.

We used PCR-based validation to examine TE insertion events in the maize inbred line MO17, while B73 served as the reference. The results for the predicted TE insertion positions show different fragment lengths between these two lines, and the sequence results support our prediction (Fig. 1b and c and Additional file 2: Figure S2).

### Non-ref TE sharing in the accessions and groups

**Fig. 2:**
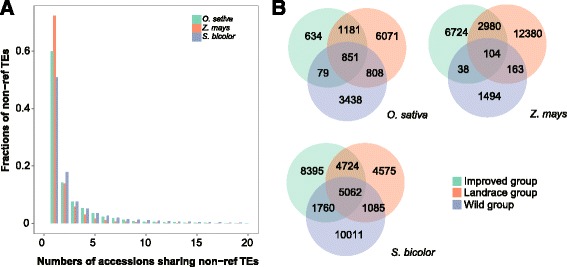
Non-ref TEs sharing in the accessions and groups. **a** Fraction of non-ref TEs present in one or shared by two or multiple accessions in rice, maize and sorghum. **b** The numbers of non-ref TEs shared between in groups

To identify the non-ref TEs shared among accessions and groups, we investigated the genome coordinates of non-ref TEs. Within 100 bp range of the insertion position of a non-ref TE, if we can identify it in two or more accessions, the non-ref TE was defined as a shared non-ref TE. In total, 7827 (60 %) rice non-ref TEs were unique among accessions, 1846 (14 %) were shared between two accessions, and 3393 (26 %) were shared between more than three accessions. In maize, 17,250 (72 %) non-ref TEs were found in only one accession, 3299 (14 %) were found in two accessions, and 3317 (14 %) were found in more than three accessions. Finally, in sorghum, 18,135 (51 %) non-ref TEs were in only one accession, 6393(18 %) were in two accessions, and 11,151 (31 %) were in more than three accessions (Fig. 2a). The majority of the identified non-ref TEs were unique, which suggested that genome polymorphisms might be best demonstrated using non-ref TEs. Furthermore, we analyzed the number of non-ref TEs shared by each accession pair, and the results showed that a high proportion of shared non-ref TEs were found in sorghum. For example, 67 % of shared non-ref TEs were between Wild#SR1000336T and Wild#SR1000339T, and 62 % were between Improved#SR1000318T and Improved#SR1000334T. These results suggest a strong phylogenetic relationship between these accession pairs (Additional file 2: Figure S3).

The non-ref TEs shared by the three groups are shown in Fig. 2b. The number of shared non-ref TEs was highest between improved and landrace groups for rice, maize and sorghum. The unique non-ref TEs were highest in the landrace groups of rice and maize and the wild group of sorghum. Considering the differences in evolutionary history for the reference genomes and the method used to discover non-ref TEs, these results suggest that differences in non-ref TEs between groups are related to the genetic relationships.

### Classification of the non-ref TEs

The classes of non-reference TEs had different preferences in rice, maize and sorghum. We classified the identified non-ref TEs into five groups, DNA, LINE, SINE, LTR and RC, according to the Repbase Update database [21]. First, we merged the non-ref TEs between accessions and compared them to the accumulated reference TEs (Table 2). Both the LTR class and the DNA class contributed the most to the accumulated reference TEs and non-ref TEs. The accumulated reference TEs had more activity in the DNA class in rice and more activity in the LTR class in maize and sorghum compared with non-ref TEs, which showed the opposite results. Second, we obtained the distribution of non-ref TEs for these accessions separately. The results showed a difference in class distribution of non-ref TEs between accessions of species, and the non-ref TE class had a different distribution compared with accumulated reference TEs (Additional file 2: Figure S4). We also compared different accessions and found the LTR and DNA of each accession with the highest number of non-ref TEs had a similar distribution.

**Table 2:** Distribution of non-ref TEs classes between accumulated TEs and non-ref TEs

Class	*O. sativa*				*Z. mays*				*S. bicolor*			
	Accumulated TEs		Non-ref TEs		Accumulated TEs		Non-ref TEs		Accumulated TEs		Non-ref TEs	
	No.	%	No.	%	No.	%	No.	%	No.	%	No.	%
DNA^a^	198156	66.98	7338	56.17	199637	16.30	7846	32.85	215449	44.53	26270	73.63
LINE	6274	2.12	43	0.33	24248	1.98	939	3.93	19208	3.97	271	0.76
LTR	69280	23.42	5472	41.88	960319	78.41	14495	60.69	235461	48.66	8863	24.84
RC	11941	4.04	83	0.64	37870	3.09	602	2.52	10395	2.15	204	0.57
SINE	9678	3.27	84	0.01	2686	0.22	3	0.01	3347	0.69	70	0.20

^a^DNA transposon

**Fig. 3:**
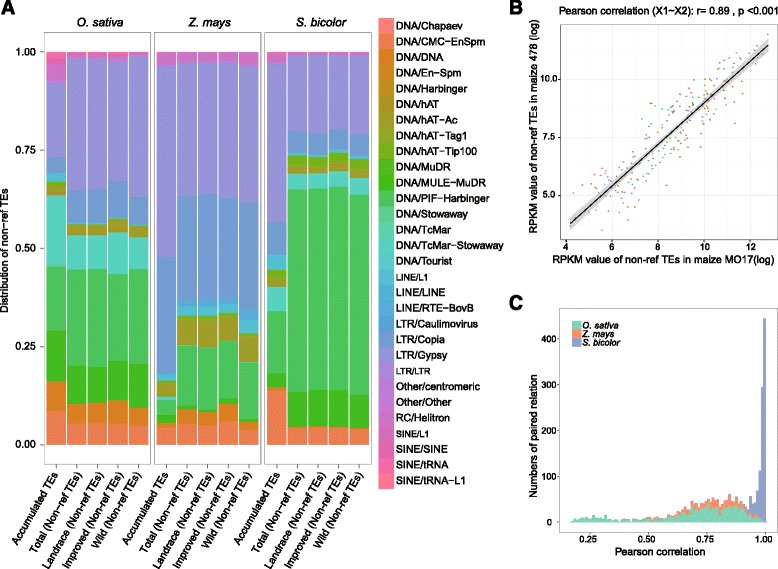
Classification of non-ref TEs in rice, maize and sorghum groups. **a** Distribution of non-ref TEs superfamilies between groups. **b** Correlation of non-ref TEs RPKM between maize_mo17 and maize_478. **c** Distribution of pearson of non-ref TEs types RPKM between two accessions

We classified TEs by superfamilies and showed that the TEs of LTR/*Gypsy* comprised 18, 48 and 40 % of the rice, maize and sorghum reference genomes, respectively, compared with the non-ref TEs, averaging 33, 34 and 19 %, respectively, in all of the accessions separately. Additionally, 16, 4 and 16 % of non-ref TEs in rice, maize and sorghum, respectively, were from the DNA/*PIF-Harbinger* class, and made up 24, 15 and 51 %, respectively, in their accumulated reference TEs (Fig. 3a). These results suggest that differences between TE classes can be observed between the superfamilies.

To further explore differences in the non-ref TEs, we compared superfamilies between accession groups. We used Student’s t-test to identify significantly different superfamilies of non-ref TEs from each group in the three species. The wild group of maize was excluded from this analysis because that group had only one accession. In rice, LINE/*L1* and RC/*Helitron* were significantly different between the improved group and the landrace group (*p* < 0.01). In maize, DNA/*DNA*, DNA/*PIF-Harbinger*, DNA/*hAT-Ac*, DNA/*hAT-Tip100*, LINE/*RTE-BovB*, LTR/*Copia* and LTR/*Gypsy* were all significantly different between the improved group and the landrace group. In sorghum, DNA/*CMC-EnSpm*, DNA/*DNA*, LTR/*Gypsy* and RC/*Helitron* were significantly different between the wild group and the improved group, and 11 superfamilies of non-ref TEs were significantly different between the wild group and the landrace group (Additional file 2: Table S1). The numbers of non-ref TE classes and superfamilies in rice, maize and sorghum are in Additional file 3: Table S5.

To discover TE differences between accessions, in cases of random sampling, the longer TE may have higher probability. We calculated the reads per kilobase per million mapped reads (RPKM) [22] for each transposon in all accessions of the three species and then calculated the Pearson correlation coefficients in pairwise comparisons. For example, the RPKM value of “Gypsy5-ZM_LTR” transposon is 4762 and 4873 in two maize accessions of Mo17 and 148; RPKM value of “LINE1-57_ZM” transposon is 122 and 76. We calculated RPKM values for each kind of non-ref TEs and their correlation coefficient between Mo17 and 148. Pearson value was 0.98, suggesting that Mo17 and 148 had similar character of non-ref TEs insertion (Fig. 3b). See all other results in Additional file 2: Figure S5. After that, the distribution of Pearson values is shown in Fig. 3c. The average Pearson correlation coefficient (PCC) of the RPKMs between accessions was 0.70, with a minimum of 0.17 and a maximum of 0.99 in rice. In maize, the average PCC was 0.77, with a minimum of 0.40 and a maximum of 0.97. In sorghum, each pairwise comparison had a PCC >0.6, with an average of 0.98, a minimum of 0.88 and a maximum of 1. Therefore, the differences in all non-ref TEs between sorghum accessions were smaller than those of rice and maize, which suggested different evolutionary histories of rice, maize and sorghum, and there have smaller genetic differences between the various accessions in sorghum.

### Chromosome distribution of non-ref TEs

**Fig. 4:**
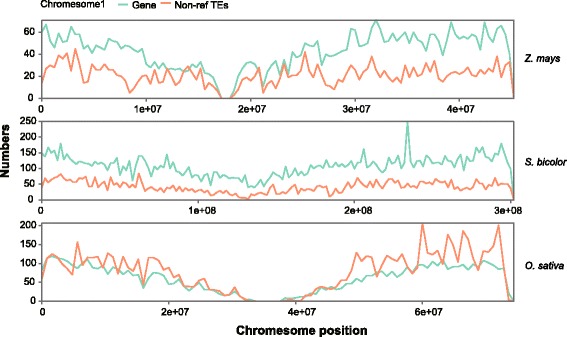
Numbers of non-ref TEs and genes in rice, maize and sorghum chromosome1

To explore the distribution of non-ref TEs, we counted the number of genes, accumulated TEs, single-nucleotide polymorphisms (SNPs) and non-ref TEs in each chromosome. We further calculated the Pearson correlation coefficient between non-ref TEs and the other three indices. Figure 4 shows the distributions of non-ref TEs and genes in chromosome 1 for rice, maize and sorghum, and the PCC are 0.61, 0.67 and 0,85, respectively. Additional file 2: Figure S6 shows the distribution of other chromosomes. Additional file 2: Table S2 shows the correlations between each pair of indices. In rice, the average PCC between non-ref TEs and gene number, accumulated TE number and SNP number were 0.12, 0.32 and 0.28, respectively, which are low correlation. In maize, non-ref TEs and gene number are positively correlated, with a PCC of 0.67, −0.01 and 0.21 were observed for the correlations between non-ref TEs and accumulated TEs and SNP number, respectively. In sorghum, non-ref TEs were positively correlated with gene number and SNP number, with average PCC of 0.88 and 0.77, respectively, and PCC of 0.53 between non-ref TEs and accumulated TEs. These results demonstrate that non-ref TEs have strong positive correlations with gene number in maize and sorghum, whereas non-ref TEs show inconsistent correlations with the other indices.

### Large effects of non-ref TEs

**Fig. 5:**
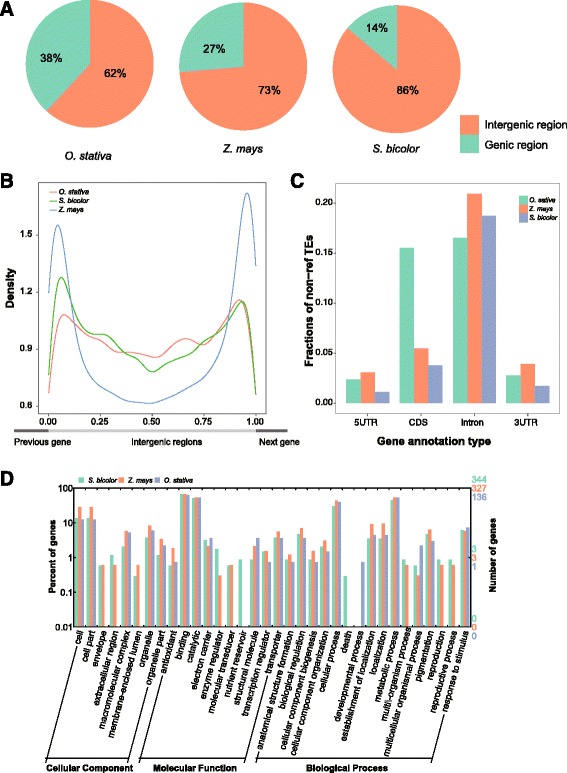
The effect of non-ref TEs in rice, maize and sorghum. **a** Distribution of non-ref TEs relative to genome annotation. **b** Density of distance from non-ref TEs to nearby gene in rice, maize and sorghum. **c** Distribution of non-ref TEs relative to gene annotation type. **d** Gene ontology analysis of genes with non-ref TEs in rice, maize and sorghum

Analysis of non-ref TE genome insertion revealed that approximately 38 % of rice non-ref TEs inserted into genic regions and 62 % inserted into the intergenic regions. In maize, the proportions of non-ref TE insertion into genic regions and intergenic regions were 27 and 73 %, respectively. The corresponding indices in sorghum were 14 and 86 %, respectively (Fig. 5a). Overall, the results indicated that the proportion of non-ref TE insertion into genic regions was highest in rice, followed by maize; the proportion for sorghum was lowest.

For non-ref TE insertion into intergenic regions, we calculated the distance between non-ref TEs and nearby genes. In rice, the average distance between two nearby genes was 9200 bp, and the average distance between non-ref TEs and nearby genes was 4491 bp. The two indices were 18,436 and 4667 bp for maize and 16,542 and 3533 bp for sorghum. The density of distance from non-ref TEs to nearby genes is illustrated in Fig. 5b. The figure clearly shows that most non-ref TEs tend to insert close to gene regions in rice, maize and sorghum, and regions closest to genes contain smaller numbers of new transposon insertions.

For non-ref TE insertion into genic regions, the ratios of the non-ref TE insertion into 5′ and 3′ untranslated regions (UTRs) are less than 5 % in rice, maize and sorghum. However, insertion into intron regions is greater than 15 %. The ratios of non-ref TE insertion into coding regions were 15.51, 5.45 and 3.76 % in rice, maize and sorghum, respectively (Fig. 5c). The proportion in rice was much higher than the proportions in maize and sorghum, suggesting that non-ref TEs in rice may have greater effects on gene function.

Generally, TE insertions alter gene expression and function. The numbers of genes with non-ref TE insertions were 4062, 4796 and 3141 in rice, maize and sorghum, respectively; the numbers of coding region insertions by non-ref TEs were 1804, 983 and 622, respectively. Additional file 2: Table S3 shows the structures of these genes compared to the reference genome annotation. Overall these results show that genes with non-ref TEs have a longer average transcript length and average CDS length and a higher average number of exons per gene compared to all of the genes in the genome.

To further investigate the effects of non-ref TE on gene function, we identified and annotated all genes with non-ref TEs in the coding region using InterProScan [23]. The results of gene annotation analysis were similar in rice, maize and sorghum. Most of these genes encoded *protein kinases*, including *protein kinase*, *catalytic domain*, *serine/threonine-/dual-specificity protein kinase*, *catalytic domain*, *tyrosine-protein kinase*, *catalytic domain*, *serine/threonine-protein kinase, active site*, *protein kinase*, *ATP binding site*, and *serine-threonine/tyrosine-protein kinase catalytic domain*. In addition to *protein kinase*, there are also some others were listed (Additional file 4: Table S6). For example, *NB-ARC*: a motif shared by plant resistance gene products and regulators of cell death in animals [24]; *Cytochrome P450*: Key players in plant development and defense [25].

Gene Ontology (GO) [26] analysis showed that function of proteins annotated in the *envelope*, *extracellular region* and *membrane-enclosed lumen* in maize and sorghum. Molecular function ontology analysis identified *enzyme regulator* and *molecular transducer* in maize and sorghum. *Nutrient reservoir* proteins were only found in sorghum. The biological process ontology analysis found proteins of *multi-organism process*, *pigmentation* and *reproduction* mainly in maize and sorghum, *depth* only in sorghum, and *developmental process* only in rice (Fig. 5d and Additional file 5: Table S7).

Biological Networks Gene Ontology (BiNGO) [27] was used to perform the enrichment analysis of GO items, such as *ATP binding*, *protein amino acid phosphorylation*, *protein kinase activity* and *apoptosis* in rice, maize and sorghum. In rice and maize, many proteins involved in *defense response* were also enriched. In addition, GO analysis in rice found cellular component enrichments for *proteolysis*, *RNA-dependent DNA replication* and *DNA integration* and molecular function enrichments for *calcium-transporting ATPase activity*, *ribonuclease H activity*, *peptidase activity* and *RNA-directed DNA polymerase activity. RNA glycosylase activity*, *isomerase activity* and *terpenoid metabolic process* were enriched in sorghum only. *Iron ion binding* was enriched in maize (Additional file 2: Figure S7). The results suggested that the genes affected by non-ref TEs were involved in multiple biological functions, and the results of the functional annotations were similar in rice, maize and sorghum.

## Discussion

### Identification non-ref TEs using resequencing data

Transposons as an important part of the plant genome, not only can regulate gene expression, gene function, but also provide important information for study of the evolution history of plants. In recent years, with the development of high-throughput sequencing technology, genome-resequencing data have been on an explosive growth trend, which includes growth in the discovery of non-ref TEs using resequencing data. Multiple studies have demonstrated the feasibility of this approach [12, 14, 17]. Our study used a modified RetroSeq workflow, adjusting some alignment methods and parameters for suitable use in genome-wide analysis of non-ref TEs in crops. A total 125 accessions of rice, maize and sorghum was used to identify novel TE insertions compared to a reference genome. The depth coverage was 6–45×, and the average numbers of non-ref TEs identified were 261, 796 and 793 for rice, maize, and sorghum, respectively. We did not find a significant correlation between the number of non-ref TEs and the depth coverage of the sequencing data. This results support the use of resequencing data to identify non-ref TEs. We found that non-ref TEs were different between accessions. We assume these differences are consistent with polymorphic variations, such as SNPs, InDels and SVs, as these DNA level changes affect polymorphisms between accessions. The investigation of non-ref TEs increases our understanding of genetic polymorphism and evolution.

### Variation of non-ref TEs among crops

**Fig. 6:**
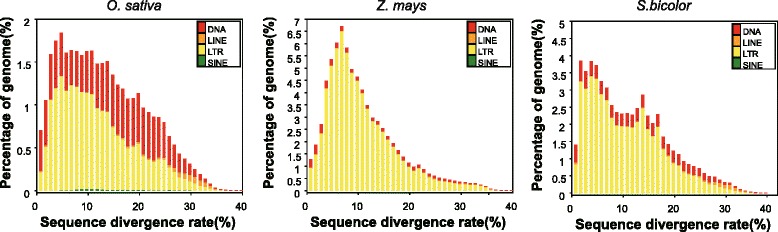
Divergence of accumulate TEs in rice, maize and sorghum

The non-ref TEs identified in rice, maize and sorghum were different. First, we identified averages of 261, 796 and 793 non-ref TEs for each accession in rice, maize and sorghum, and the NPSPDs were 0.28, 4.35 and 0.89, respectively. So the non-ref TEs number is obviously different among species, which of rice is far less than that of maize and sorghum. Second, our analysis shows an inverse relationship for TE classes between non-ref TEs and accumulated TEs. In rice, most accumulated TEs belongs to DNA class, but LTRs were the most common identified in non-ref TEs. By contrast, for maize and sorghum, the LTR proportion was highest in accumulated TEs and lower in non-ref TEs. We also analyzed the divergence of accumulated TEs. The results in rice show that the average divergence rate was 17 %, and the divergence rates in maize and sorghum were both 15 %. Moreover, DNA class has a greater divergence rate than LTR in rice (Fig. 6). We speculate that the higher divergence in rice influences the alignment process, resulting in more false-negative results and fewer DNA transposon identifications. This possibility may also explain our findings that LTR transposons are more active in maize and sorghum and DNA transposons in rice are more active in maize and sorghum. At last, non-ref TEs difference among species is related to genome stability. Rice genome is smaller and more conservative than maize and sorghum, which may be related to their growth environment and evolution history.

Differences in transposons in the genome occur not only between species but also between groups. We divided the accessions of rice, maize and sorghum separately into three groups: wild, improved and landrace. First, we analyzed the numbers of non-ref TEs between different groups. The average numbers of non-ref TEs in improved, landrace and wild groups of rice were 249, 307 and 518, respectively, 1641, 679 and 1798 in maize, respectively, and 999, 858 and 2563 in sorghum, respectively. These results indicate that there are more non-ref TEs in the wild group than in the improved and landrace groups in rice, maize and sorghum. The results of the NPSPD analysis were similar (Table 1). Because non-ref TEs are defined as TEs that are not in the reference genome but in other accession genomes, we note that accessions that are closely related to the reference genome may be identified with fewer non-ref TEs. By contrast, increased genetic distance would result in more non-ref TEs. The cultivar sequencing of rice (Japonica), maize (B73) and sorghum (BTx623) provide reference genomes, so these reference genomes are more distantly related to the wild group and more closely related to their domestication and improvement processes. Second, we compared the superfamilies of non-ref TEs. Significant superfamily differences were observed among the groups. Identifying the source of these differences requires further analysis; however, we speculate that these differences are also related to evolutionary history, genetic relationships between accessions and the distance from accessions to the reference genome.

### Non-ref TE insertions are not random events

The four following lines of evidence suggest that non-ref TE insertions are not random events:

1. **Positive correlation between non-ref TEs and gene density.** Other researchers are also concerned about the relationship between genes and transposons. In Arabidopsis, distribution analysis of accumulated TEs suggests a negative correlation between gene and TE density [28, 29]. This association is also found in rice, where investigation of non-LTR-RTs (Non-long terminal repeat retrotransposons) and DNA transposons revealed a negative correlation between gene densities [30, 31]. We analyzed the chromosomal distributions of non-ref TEs and genes in sorghum and maize, and found that they were strongly correlative, and the respective mean PCC were 0.88 and 0.67. Our discovery of this relationship between non-ref TEs and gene number is novel. The results suggest that the TEs in the region near a gene have high activity, whereas accumulated TEs are more stable. Moreover, the position of non-ref TE insertion in sorghum was positively related to SNP loci; this relationship is also clearly shown for accumulated TEs in the human genome [32, 33]. Presumably, these non-ref TEs are an important source of SNPs, and in rice and maize but not sorghum, non-ref TEs have a smaller contribution to SNPs.
  The number and distribution of non-ref TEs in rice is different from those of maize and sorghum, meanwhile, the correlation coefficient between non-ref TEs and gene number in rice is far less than those of maize and sorghum. The possible reasons are as follows. 1) the total gene number in rice, maize and sorghum genomes is similar to each other. However, the genome size of rice is far less than sorghum and maize. So the rice genome included fewer TEs. 2) Previous reports showed that rice genome is more conservative [34, 35]. It was speculated that the TE activity is lower than other grasses, such as maize and sorghum, which causes small TE difference among rice accessions. So we identified fewer non-ref TEs in rice. Comparing the gene and non-ref TEs distribution among rice, maize and sorghum, the similar total gene number with less accumulated and non-ref TEs of rice may results in the weakly correlated between gene number and non-ref TE.
2. **Non-ref TEs are often located at flanking regions of genes.** The analysis of distance between non-ref TEs and nearby genes found that non-ref TE insertions tended to be close to intergenic regions, keeping their distance from upstream and downstream genes. The distribution of miniature inverted repeat transposable elements (MITEs) in regions near genes for rice was also confirmed [36]. This TE activity located at regions flanking genes can result in complex rearrangements that can affect gene regulation [37, 38]. These results suggest that location biases in non-ref TE insertion may play important roles in gene regulation.
3. **Non-ref TEs are often located in introns.** TEs that insert into introns generally have a greater chance of survival because these insertions are less visible to natural selection. Moreover, TE insertions into introns can affect gene regulation in surprising ways [11, 37, 39]. In our analysis of non-ref TE insertion position, the ratio of transposons that inserted into intron regions was greater than 15 %, and the ratio of rice non-ref TE insertion into CDS regions was 15.51 %, compared with 5.45 and 3.76 % in maize and sorghum, respectively. The proportion of non-ref TE insertion into intron regions was much higher than the proportions of insertion into CDS regions, and the proportion of non-ref TE insertion into CDS regions in rice was much higher than the proportions of insertion into maize and sorghum. These results suggest two possibilities. First, natural selection negatively influences detection of TE insertion in exon regions. TE insertions often leads to disrupting the structure and function of genes. After a long time evolutionarily speaking, they will become so diverged that they are no longer identifiable. Second, transposon insertion may occur with a preference to avoid coding regions, or coding region protective mechanisms render TE insertion difficult. Additionally, the transcriptional state of DNA influences DNA structure, which may affect TE insertion. Assuming efficient transposon insertion, such insertions likely occur primarily during the process of transcription. In agreement with this mechanism, in rice, maize and sorghum, genes with non-ref TEs have longer average transcript and CDS lengths and higher average exon numbers per gene.
4. **Non-ref TEs response to stress.** The responses of genomes to stress by transposons was first suggested by McClintock [40]. Two approaches can be used to test this hypothesis. The first involves stress exposure to genetically controlled organisms [41–44]. The second approach involves analysis of natural populations of the same species living in different conditions [45]. Here, we analyzed the functions of genes that are affected by non-ref TE insertion. Although the identified non-ref TEs number in rice is far less than maize and sorghum, the results of gene function annotation and classification are consistent. Interpro results showed that most affected genes encoded proteins annotated as *protein kinases* which involved in many aspects of cellular regulation and metabolism [46]. Additional, some affected genes were annotated as *NB-ARC* and *Cytochrome P450* which involved in plant resistance gene and defense. GO analysis showed that affected genes are functionally different. The GO enrichment analysis identified affected genes encoding proteins that have ATP-binding sites, amino acid phosphorylation sites, and protein kinase activity, along with biological processes related to cell apoptosis in rice, maize and sorghum. In addition, affected genes in rice and maize included functional enrichments for defense response processes. These results demonstrate that the functions of genes affected by non-ref TE insertion are highly similar in the three crops. Protein phosphorylation alters both protein structure and activity to influence the transmission process of information in a cell. Through a series of protein phosphorylation and dephosphorylation steps, plant cells transmit intracellular signals to generate an appropriate response to extracellular stimuli. Results of the functional enrichment analysis suggest that plant cells experiencing stressful external stimulation activate intracellular kinase activities to affect protein ATP-binding sites. Autophosphorylation may follow, and then phosphate group transfer to other proteins to amplify the signal cascade regulating downstream gene expression, which leads to either cell apoptosis or the promotion of defense reactions to increase crop resilience. Thus, in the event of plant environmental stress, gene protection mechanisms are activated, and transposons may be inserted into specific gene regions to maintain defensive intracellular signal transduction for improving crop adaptability to adverse environmental conditions. In this way, transposon insertion may play an important role in plant adaptive evolution.

## Conclusions

Transposable elements (TEs) are a major component of plant genomes, but their characteristics of various accessions is not clear. We present the genome-wide identification and characterization of non-reference transposable elements in rice, maize and sorghum using resequencing data. Our results show that the non-ref TE class has different preferences in rice, maize and sorghum. The non-ref TEs have a strong positive correlation with gene number and have a bias toward insertion near genes, and also with a preference for avoiding coding regions. The genes affected by non-ref TE insertion were functionally enriched for stress response mechanisms. Suggest that transposon insertion is not a random event and it makes genomic diversity to plays a major role in intraspecific adaption and evolution of crops. It provides new insight into the evolution of transposons and their role in plant evolution. In the near future, more plant genomics data should analysis to improve understanding of the transposon evolution and how their insertion may have influenced the variation between accessions.

## Methods

### Datasets

Maize resequencing data were acquired from a project deposited in the NCBI Short Read Archive with accession number SRA010130 [47]. This project generated resequenced data from a group of six elite maize inbred lines. Another maize resequencing dataset was acquired from the NCBI Short Reads Archive under the accession number SRA051245 [16]. The data from this maize HapMapV2 study had a depth coverage that ranged from 4X to 30X. We only used landrace lines sequenced at the same facility. The rice resequencing data acquired from the NCBI Short Read Archive under accession number SRA023116 [17], had 50 accessions in total, which included 40 cultivated accessions and ten accessions of wild progenitors with >15X raw data coverage. The acquired sorghum resequencing data had 16-45X raw data coverage of 45 sorghum lines [18].

The transposon data from the three species studied were extracted from the Repbase Update database at www.girinst.org/repbase/ [21]. The Repbase Update (RU) database contains prototypic sequences representing repetitive DNA from different eukaryotic species.

The B73 sequences of the maize reference genome were obtained from the Maize Genome Sequencing Project AGPv2, which was the first draft assembly of the maize genome released in 2010. We used the maize gene annotation from the 5b.60 release of the maize Genome Sequencing Project based on the AGPv2 assembly [4]. The International Rice Genome Sequencing Project (IRGSP) genome sequence (build 5) was used as the rice reference genome. Accordingly, rice annotation information used the 2009-01-MSU gene set [48]. The sorghum reference genome used was the DOE-JGI (sbi1), and the Sbi1.4 gene set was used for gene annotation [3].

### Identifying non-ref TEs

First, we used BWA (0.6.2-r126) [49] to map the next-generation sequencing reads against the reference genome, and then we used the ‘bwa sampe’ to generate paired reads alignment in SAM format. Second, we used SAMTOOLS (Version: 0.1.18) [50] to sort the alignments and then used the command ‘samtools merge’ to merge the alignments from different sequence lanes.

To identify candidate non-ref TEs with read pair support, we used a modified RetroSeq [19] workflow, adjusting some alignment methods and parameters for suitability in genome-wide analysis of non-ref TEs in crops. First, we checked the bam file, and the alignments with duplicate reads or a mapping quality <30 were discarded. We kept mapped reads and unmapped mate reads for further analysis. Second, we used the unmapped mate reads in a BLAST query search [51] against the TE library. Matches with >80 % identity and alignment length >36 in the 500 bp beginnings or 500 bp ends of sequences in the TE library were accepted as candidate non-ref TEs.

We clustered the reads into fwd and rev clusters in 4000-bp regions. Only an average length of a region <200 bp and a minimum number of reads >5 was considered for non-ref TE identification. Finally, we compared candidate read positions against the accumulated TEs and filtered the results that overlapped with the reference (Additional file 2: Figure S1).

We used PCR to validate a sample of non-ref TE insertions in the MO17 maize inbred line. Primers were designed to the predicted non-ref TE insertion site. Comparisons of amplicon sizes between references B73 and MO17 were used to determine insertions of predicted non-ref TEs. The PCR products were also sequenced for comparison to further establish the presence of an insertion event (Fig. 1b and Additional file 2: Figue S2).

### Genome-wide characterization analysis of non-ref TEs

Pearson correlation coefficient was calculated between sequencing depth and the number of non-ref TEs of all accessions. The numbers of non-ref TEs in each accessions were obtained after the identifying steps. We clustered the non-ref TEs inserted position in 100 bp regions of all accessions for identifying accessions or groups sharing a common non-ref TE.

Classification using the Repbase database divided transposons into classes and subclasses. There were six classes: DNA transposon, LINE, SINE, LTR, RC and Other. The subclasses mainly included LTR/*Copia*, LTR/*Gypsy*, DNA/*CMC-EnSpm*, DNA/*MULE-MuDR*, LINE/*L1* and RC/*Helitron*, etc. To explore the superfamilies differences between groups, student’s t-test was used to identify significantly different superfamilies of non-ref TEs from each group in the three species. RPKM was calculated as the total number of short sequences mapped to transposon type divided by the total number of short sequences mapped to the transposon database (million) and the length of transposon type (KB) [22]. We used all of these transposon’s RPKM value to calculate the Pearson correlation coefficients of all accessions in pairwise.

The numbers of genes, SNPs, transposons, and non-ref TEs were calculated for each reference genome chromosome. For rice, the calculation window size was 400 KB, with a sliding window size of 200 KB. The maize window size was 2 MB, with a sliding window size of 1 MB. The calculation window size in sorghum was 600 KB, with a sliding window size of 300 KB. To calculate gene numbers on each chromosome, the gene start site was used, and measurements were transformed into log2 values for drawing.

We obtained the protein sequences of genes affected by non-ref TEs for a BLASTP search against TrEMBL [52], KEGG [53] and SwissProt [54] databases. The e-value was set to 1-5e, and we only retained the best match for each protein. For structure domain and motif characterization, we used InterProScan to search Pfam, PRINT, PROSITE, ProDom and SMART databases. Homologous protein sequences found after InterProScan analysis were used for GO analysis. We used WEGO [55] for GO mapping and BINGO for GO enrichment analysis.

## Abbreviations

CDS, coding sequence; GO, gene ontology; LINEs, long interspersed elements; LTRs, long terminal repeats; non-ref TEs, non-reference transposable elements; NPSPD, average number of non-ref TEs per sample per depth; PCC, pearson’s correlation coefficient; RC, rolling-circle transposon; RPKM, reads per kilobase per million mapped reads; SINEs, short interspersed elements; SNP, single-nucleotide polymorphisms; TE, transposable element; UTRs, untranslated regions

## Additional files

Additional file 1: Table S4. Numbers of non-ref TEs and reads depths in rice, maize and sorghum accessions. (XLS 53 kb) Additional file 2: Supplemental Material. This file contains **Tables S1–S3** and all the **Figures S1–S7**. (PDF 1647 kb) Additional file 3: Table S5. Classification of non-ref TEs in crops. (XLS 62 kb) Additional file 4: Table S6. Top 30 represented InterPro terms from the InterProScan annotations. (XLS 41 kb) Additional file 5: Table S7. GO enrichment of genes with non-ref TEs in rice, maize and sorghum. (XLS 55 kb)
