# Supplementary material for: Genome-wide characterization of non-reference transposons in crops suggests non-random insertion
Source: BMC Genomics. 2016 Aug 2;17:536. doi: 10.1186/s12864-016-2847-3 (PMC4971691; doi:10.1186/s12864-016-2847-3)

Supplemental Table 1. Significantly different of non-ref TEs in superfamilies between accession groups in rice, maize and sorghum. 2

Supplemental Table 2. Correlations between numbers of non-ref TEs, accumulated TEs, genes and SNPs in rice, maize and sorghum. 3

Supplemental Table 3. Statistics of genes structure in rice, maize and sorghum......................................4

Supplemental Figure 1. Overview of non-ref TEs calling. 5

Supplemental Figure 2. Non-ref TEs insert in MO17 accession of maize (DNA-4_ZM). 5

Supplemental Figure 3. Non-ref TEs shared by each two accessions. 6

Supplemental Figure 4. Distribution of non-ref TEs class in maize and sorghum accessions. 7

Supplemental Figure 5. Correlation of non-ref TEs RPKM between maize_Mo17 and other accessions. 8

Supplemental Figure 6. Distribution of non-ref TEs, accumulated TEs, genes and SNPs in rice, maize and sorghum chromosomes. 9

Supplemental Figure 7. Gene ontology enrichment analysis of effected genes by non-ref TEs in rice, maize and sorghum. 10

Supplemental Table 1. Significantly different of non-ref TEs in superfamilies between accession groups in rice, maize and sorghum. The average number of different superfamilies of non-ref TEs was counted in all accessions, then we used Student's t-test to identify significantly different superfamilies between groups.

**Species**	**Superfamily**	**Mean**[a]		**Ratio**	***F***	***P***[b]
O. sativa		Improved	Landrace			
	LINE/*L1*	1.00	3.17	0.32	3.27	2.34E-03
	RC/*Helitron*	1.09	3.59	0.30	3.24	2.52E-03
Z. mays		Improved	Landrace			
	DNA/*DNA*	95.17	35.87	2.65	10.04	6.74E-11
	DNA/*PIF-Harbinger*	312.67	167.48	1.87	5.89	1.40E-06
	DNA/*hAT-Ac*	123.83	64.35	1.92	6.16	7.00E-07
	DNA/*hAT-Tip100*	15.00	7.00	2.14	5.65	2.71E-06
	LINE/*RTE-BovB*	32.67	17.22	1.90	6.78	1.40E-07
	LTR/*Copia*	560.83	284.04	1.97	6.70	1.71E-07
	LTR/*Gypsy*	780.50	369.61	2.11	5.13	3.67E-03
S. bicolor		Wild	Improved			
	DNA/*CMC-EnSpm*	152.00	99.60	1.53	3.32	2.76E-03
	DNA/*DNA*	13.86	7.30	1.90	4.50	1.38E-04
	DNA/*MULE-MuDR*	331.00	204.55	1.62	2.99	6.16E-03
	DNA/*PIF-Harbinger*	2115.57	1256.05	1.68	3.64	1.26E-03
	DNA/*TcMar-Stowaway*	196.71	101.95	1.93	4.19	3.01E-04
	DNA/*hAT-Tag1*	23.00	11.10	2.07	3.45	2.02E-03
	LTR/*Copia*	198.14	121.85	1.63	3.63	1.28E-03
	LTR/*Gypsy*	635.29	415.20	1.53	3.46	1.94E-03
	LTR/*LTR*	9.86	5.00	1.97	3.08	5.00E-03
	RC/*Helitron*	24.14	12.70	1.90	4.21	2.84E-04
	SINE/*tRNA*	9.29	5.00	1.86	2.89	7.88E-03
		Wild	Landrace			
	DNA/*CMC-EnSpm*	152.00	95.44	1.59	3.81	9.00E-04
	DNA/*DNA*	13.86	6.44	2.15	5.46	1.74E-05
	LTR/*Gypsy*	635.29	398.56	1.59	4.94	5.70E-05
	RC/*Helitron*	24.14	10.17	2.37	5.76	3.57E-06

[a]Mean number of non-ref TEs in superfamilies between groups. [b]Student's *t* test.

Supplemental Table 2. Correlations between numbers of non-ref TEs, accumulated TEs, genes and SNPs in rice, maize and sorghum. The numbers of genes, SNPs, accumulated TEs and non-ref TEs were counted with sliding windows (For rice, the calculation window size was 400 KB, with a sliding window size of 200 KB. The maize window size was 2 MB, with a sliding window size of 1 MB. The calculation window size in sorghum was 600 KB, with a sliding window size of 300 KB.) for each chromosome. Then we calculated the Pearson correlation coefficient between any two indices. Pearson's correlation coefficient absolute value greater than 0.6 were marked as red.

**Species**	**Chromosome**	**Pearson correlation**					
		Non-ref TEs vs Genes	Non-ref TEs vs accumulated TEs	Non-ref TEs vs SNPs	Accumulated TEs vs SNPs	Genes vs accumulated TEs	Genes vs SNPs
O. sativa	chr1	0.61	0.33	0.35	0.82	0.24	0.13
	chr2	0.3	0.32	0.47	0.78	-0.09	-0.15
	chr3	0.33	0.36	0.26	0.83	-0.04	-0.21
	chr4	0.22	0.18	0.3	0.73	-0.26	-0.48
	chr5	0.3	0.43	0.3	0.73	0.09	-0.24
	chr6	0.22	0.56	0.43	0.77	0.18	-0.17
	chr7	0.44	0.29	0.18	0.61	0.14	-0.28
	chr8	0.07	0.36	0.42	0.57	0.15	-0.33
	chr9	0.02	0.42	0.47	0.78	0.11	-0.21
	chr10	-0.08	0.42	0.54	0.71	0.23	-0.19
	chr11	0.44	0.39	0.32	0.86	0.27	0.12
	chr12	0	0.37	0.38	0.61	0.11	-0.4
Z. mays	chr1	0.67	0.13	0.09	0.67	0.08	-0.12
	chr2	0.74	-0.02	0.04	0.67	-0.01	0.01
	chr3	0.78	0.23	0.49	0.62	0.27	0.46
	chr4	0.7	0.12	0.39	0.46	0.15	0.24
	chr5	0.8	-0.1	0.28	0.52	-0.06	0.31
	chr6	0.66	0.2	0.34	0.69	0.2	0.22
	chr7	0.66	0.21	0.36	0.5	0.2	0.26
	chr8	0.51	0.18	0.35	0.55	0.19	0.41
	chr9	0.48	0.16	0.17	0.57	0.2	0.11
	chr10	0.86	0.19	0.07	0.8	0.22	0.09
S. bicolor	chr1	0.85	0.54	0.82	0.83	0.55	0.8
	chr2	0.89	0.43	0.89	0.6	0.59	0.81
	chr3	0.82	0.48	0.91	0.52	0.64	0.72
	chr4	0.8	0.41	0.89	0.55	0.57	0.67
	chr5	0.7	0.4	0.87	0.56	0.59	0.58
	chr6	0.85	0.21	0.87	0.35	0.41	0.78
	chr7	0.78	0.42	0.9	0.56	0.66	0.71
	chr8	0.64	0.25	0.91	0.38	0.61	0.63
	chr9	0.77	0.39	0.9	0.52	0.56	0.68
	chr10	0.9	0.58	0.83	0.86	0.56	0.82

Supplemental Table 3. Statistics of genes structure in rice, maize and sorghum.

Genes structure	*O. sativa*			*Z. mays*			*S. bicolor*		
	Total genes[a]	Gene with non-ref TEs[b]	CDS with non-ref TEs[c]	Total genes[a]	Gene with non-ref TEs[b]	CDS with non-ref TEs[c]	Total genes[a]	Gene with non-ref TEs[b]	CDS with non-ref TEs[c]
Gene Number	42088	4062	1804	110185	4796	983	34080	3141	622
Average transcript length (bp)	3007.44	4759.9	4316.65	2227.91	7561.85	4817.21	2862.72	4899.22	3161.24
Average CDS length (bp)	957.28	1884.68	2342.55	630.71	1212.23	1131.67	1161.1	1421.12	1483.25
Average exon per gene	3.77	6.17	5.45	2.8	6.53	5.31	4.3	6.6	4.71
Average exon length (bp)	253.74	305.66	429.56	225.1	185.69	213.03	270.02	215.28	314.87
Average intron length (bp)	739.42	556.57	443.28	886.39	1148.6	854.66	515.64	620.93	452.21

[a] Total genes from genome annotation. [b] Genes with non-ref TEs. [c] Genes with non-ref TEs in coding sequence region.

Supplemental Figure 1. Overview of non-ref TEs calling. Briefly, we first mapped the next-generation sequencing reads against the reference genome, and selected mapped reads and unmapped mate reads for further analysis. Then we made blast against the TE library using the unmapped mate reads as query sequence. These positions of paired end reads cluster were accepted as candidate non-ref TEs insertion.

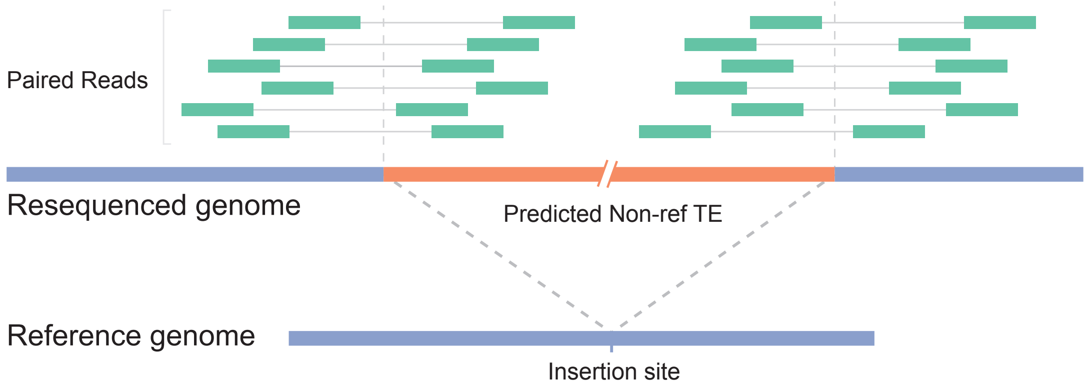

Supplemental Figure 2. Non-ref TEs insert in MO17 accession of maize (DNA-4_ZM). PCR-based validation of non-ref TEs insertion in MO17, the sequence marked as red was the transposon insertion in MO17 compare to B73.

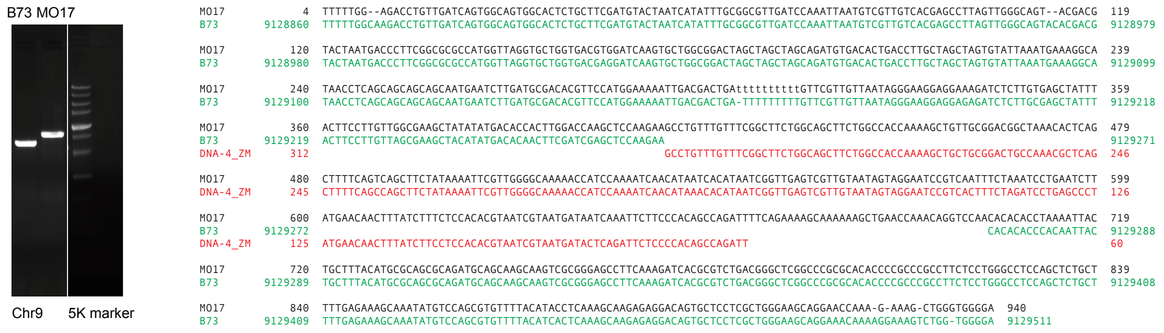

Supplemental Figure 3. Non-ref TEs shared by each two accessions. Within 100bp range of the insertion position of a non-ref TE, if we can identify it in two or more accessions, the non-ref TE was defined as a shared non-ref TE. We analyzed the number of non-ref TEs shared by each accession pair, and the result shows that a high proportion of shared non-ref TEs were found in sorghum.

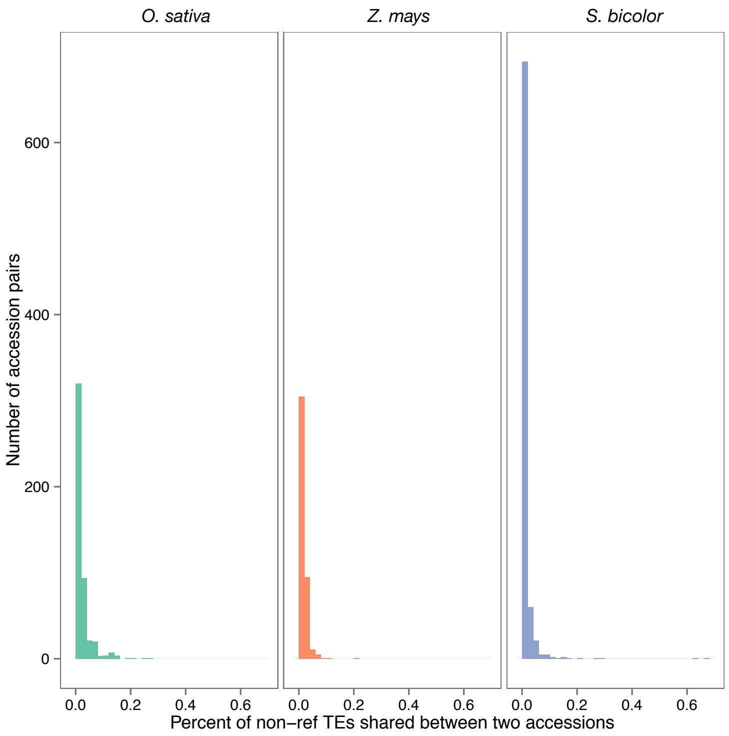

Supplemental Figure 4. Distribution of non-ref TEs class in maize and sorghum accessions. A, *O. sativa*; B, *Z. mays*; C, *S. bicolor*

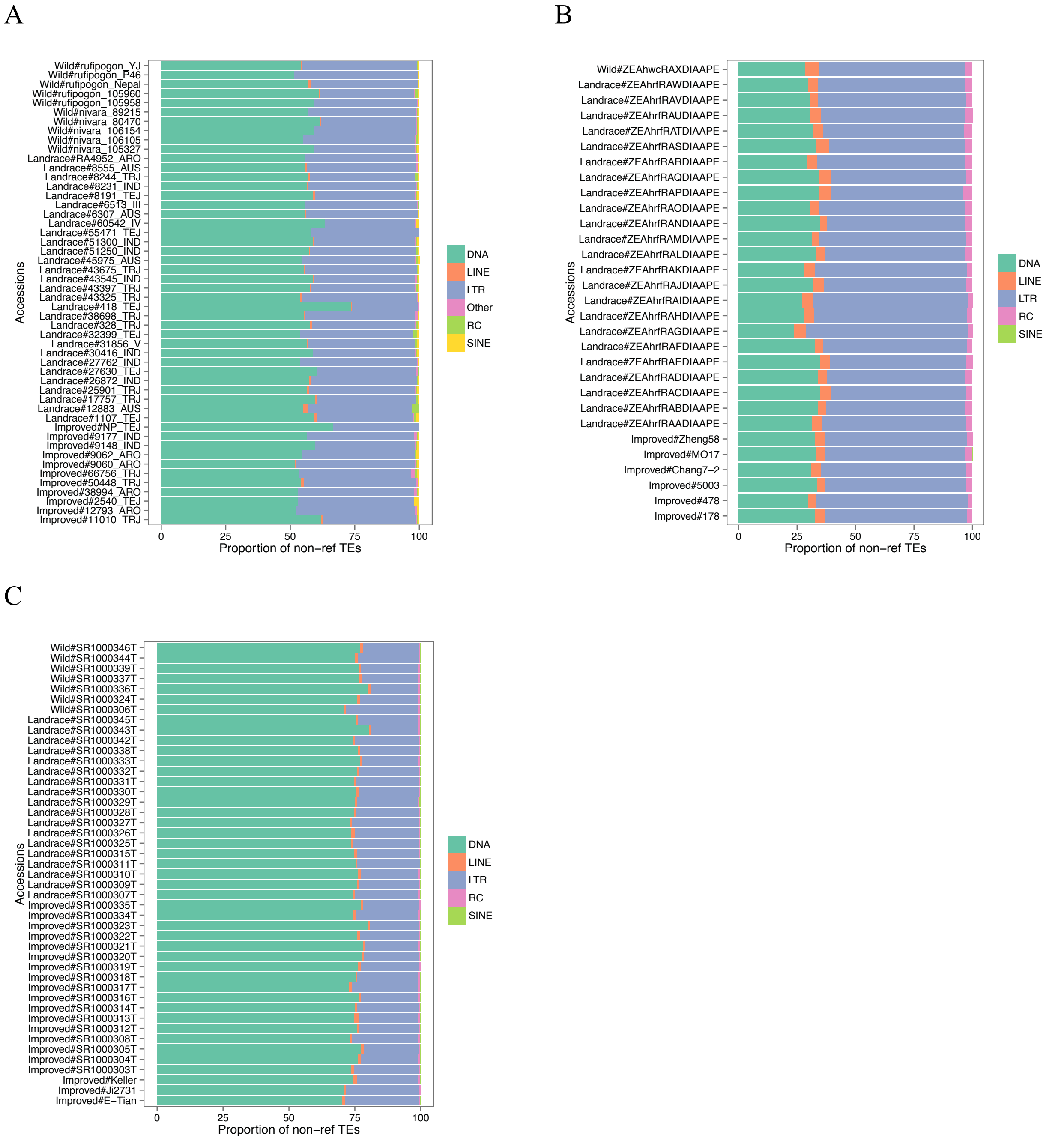

Supplemental Figure 5. Correlation of non-ref TEs RPKM between maize_Mo17 and other accessions. The reads per kilobase per million mapped reads (RPKM) for each transposon in all maize accessions were calculated, then we compared RPKM between MO17 and other accessions, and calculated the Pearson correlation coefficients.

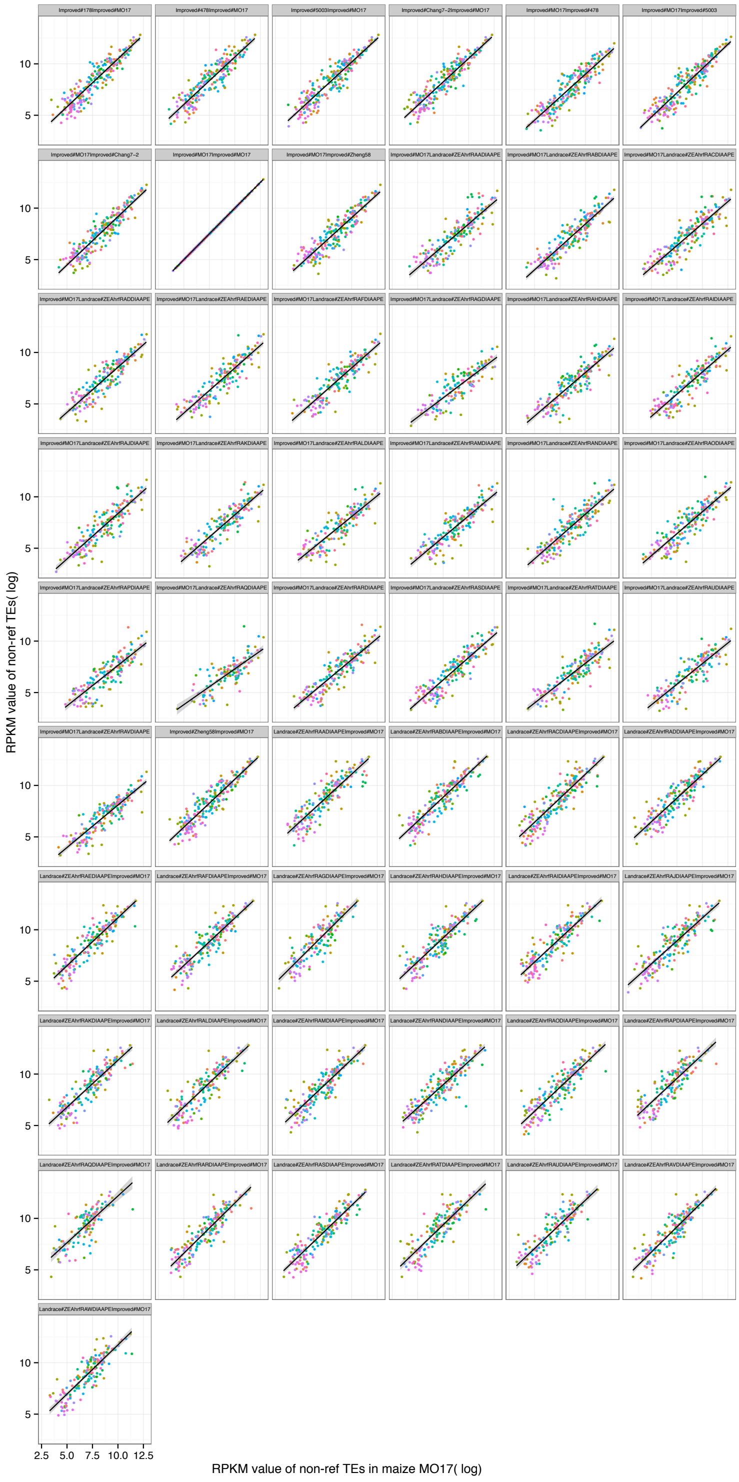

Supplemental Figure 6. Distribution of non-ref TEs, accumulated TEs, genes and SNPs in rice, maize and sorghum chromosomes. A, *O. sativa*; B, *Z. mays*; C, *S. bicolor.*

A

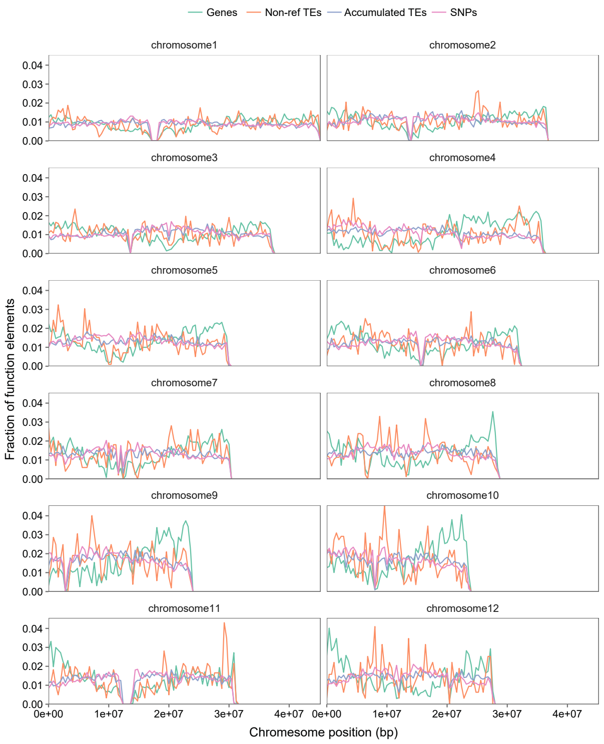

B

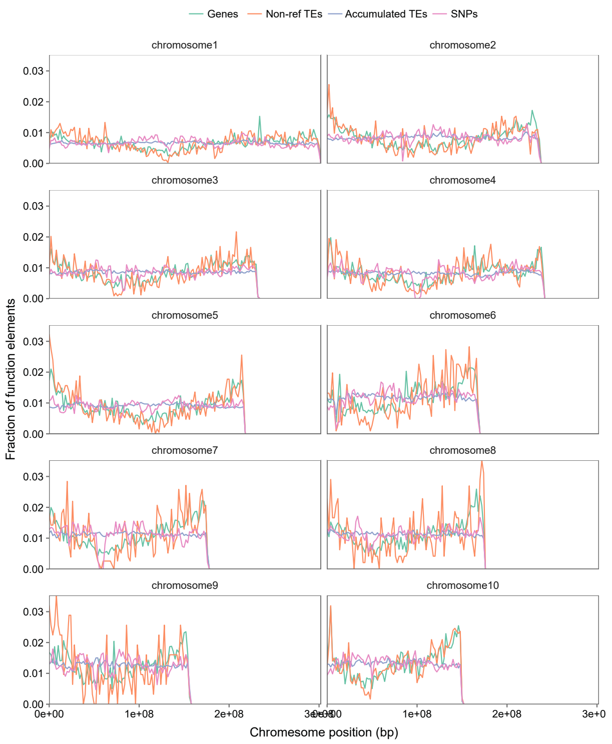

C

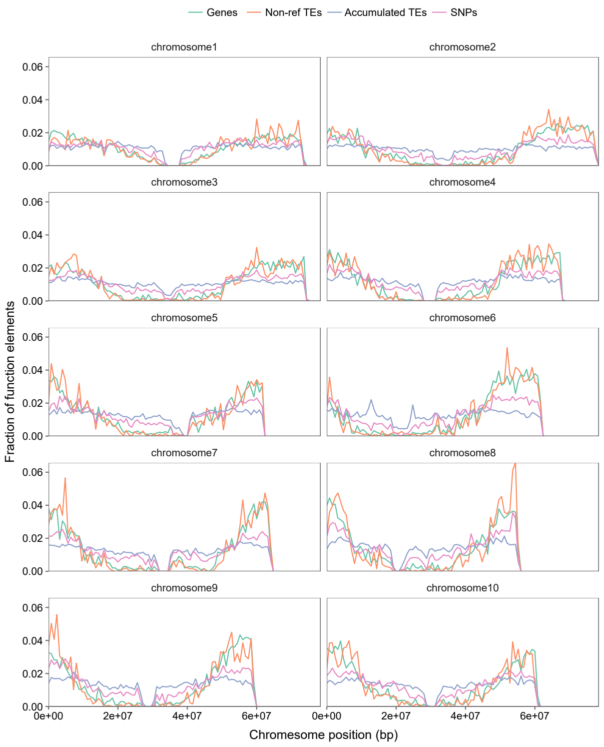

Supplemental Figure 7. Gene ontology enrichment analysis of effected genes by non-ref TEs in rice, maize and sorghum. Networks of over-represented GO items in the gene sets with non-ref TEs insertion are show. The color of the node represents the (corrected) *p*-value. Cytoscape and BiNGO were used to construct the networks. A, *O. sativa*; B, *Z. mays*; C, *S. bicolor.*

A

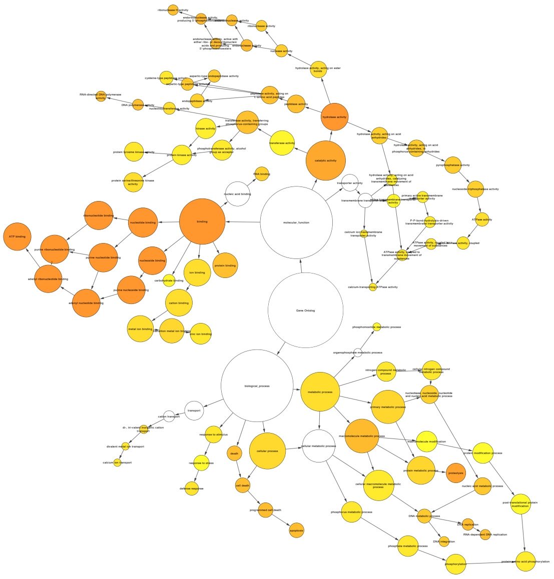

B

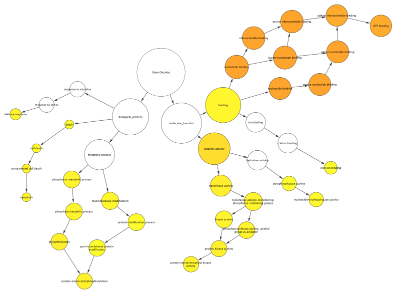

C

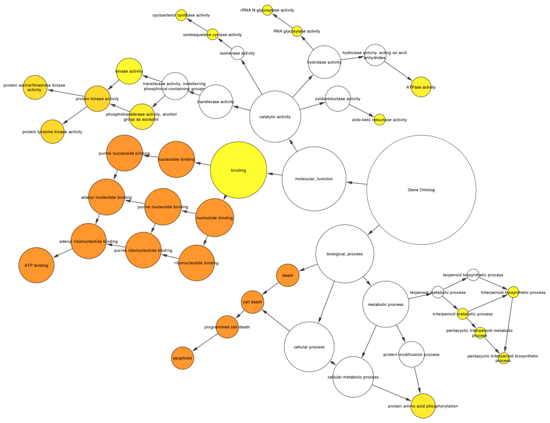

Supplement: Additional file 2: — Supplemental Material. This file contains Tables S1–S3 and all the Figures S1–S7. (PDF 1647 kb) [file 12864_2016_2847_MOESM2_ESM.pdf]
